# Exploring the association between cognitive performances, early life traumas, and lifetime suicide attempts in Major Depressive Disorder: an analysis of the PROMPT study

**DOI:** 10.1192/j.eurpsy.2026.12067

**Published:** 2026-07-31

**Authors:** P. Paribello, V. T. Wahner, M. Pinna, M. Contu, C. Pisanu, A. Squassina, A. Minelli, M. Manchia, B. T. Baune, PROMPT consortium

**Affiliations:** 1 University of Cagliari, Cagliari, Italy; 2 Dept of Psychiatry, University of Münster, Münster, Germany; 3 University of Brescia; 4 IRCCS San Giovanni di Dio Fatebenefratelli Center, Brescia, Italy; 5 University of Münster, Münster, Germany; 6 Florey Institute of Neuroscience and Mental Health; 7 University of Melbourne, Melbourne, Australia

## Abstract

**Introduction:**

Suicidal behaviour in Major Depressive Disorder (MDD) arises from the interplay of cognitive, environmental, and biological factors. Cognitive performances may modulate vulnerability, yet longitudinal evidence remains limited.

**Objectives:**

To test whether cognitive performances assessed with the *Brief Assessment of Cognition in Affective Disorders* (BAC-A) are associated with lifetime suicidality, adjusting for early trauma and clinical covariates, within the European multicentre *Personalised Prognostic Models in Depression* (PROMPT) study.

**Methods:**

We analysed 292 adults with moderate-to-severe MDD (Cagliari n = 102, Münster n = 119, Poznań n = 71), aged 18–65 years, diagnosed per DSM-5 criteria (Montgomery–Åsberg Depression Rating Scale ≥ 20). BAC-A was administered at baseline, week 8, and week 12. Suicidality was measured using the Columbia Suicide Severity Rating Scale (C-SSRS), selecting items 1 (passive ideation), 3 (active ideation without plan), and 5 (lifetime attempts). Linear mixed-effects models tested non-affective recall as the dependent variable, including suicidality, timepoint, and their interaction as fixed effects, and adjusting for age, sex, education, depression severity, site, early trauma, substance use, ECT or ketamine treatment, and current use of psychotropics. Holm’s correction addressed multiple comparisons.

**Results:**

Table 1 presents the main demographic and clinical characteristics of lifetime active suicide ideation (n = 292 with available data for analyses). Participants with lifetime active ideation (C-SSRS item 3) showed significantly lower non-affective recall (estimate = –1.02, Holm-corrected p = 0.042), independent of depression severity and trauma. Age, education, site, and antipsychotic use predicted overall cognition, while no time interactions emerged.

**Table 1.** Main demographic and clinical characteristics by C-SSRS item 3 lifetime status Table 1. long description.

Variable	No (n = 174)	Yes (n = 118)
Age (years), median [IQR]	52 [37–62]	41 [29–55]
Female sex %	62.6	54.2
Education ≥ High School %	59.0	62.1
MADRS score, median [IQR]	22 [19–26]	27 [23–33]
CTQ total, median [IQR]	40 [32–52]	47 [35–59]
Antipsychotic use %	26.4	51.7
TRD current episode %	32.2	46.6
Recruitment site (Münster/Cagliari/Poznań %)	24.7/52.3/23.0	64.4/9.3/26.3

Figure 1, 2 and 3. Error margins plot for the association of BAC-A Non-affective recall over the three selected timepoints, divided based on CSSR-S status and recruitment sites.

**Image 1:**
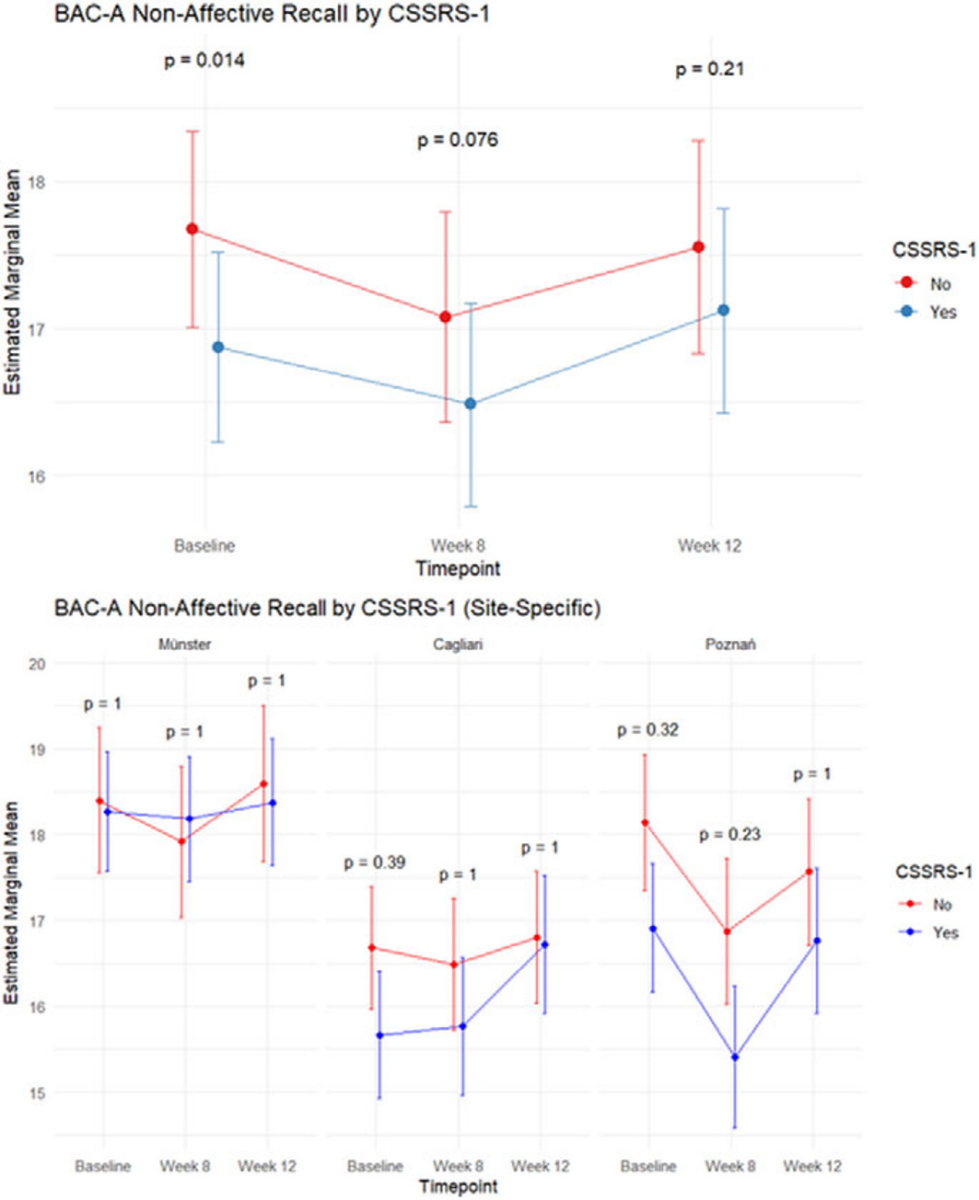
Image 1: long description.

**Image 2:**
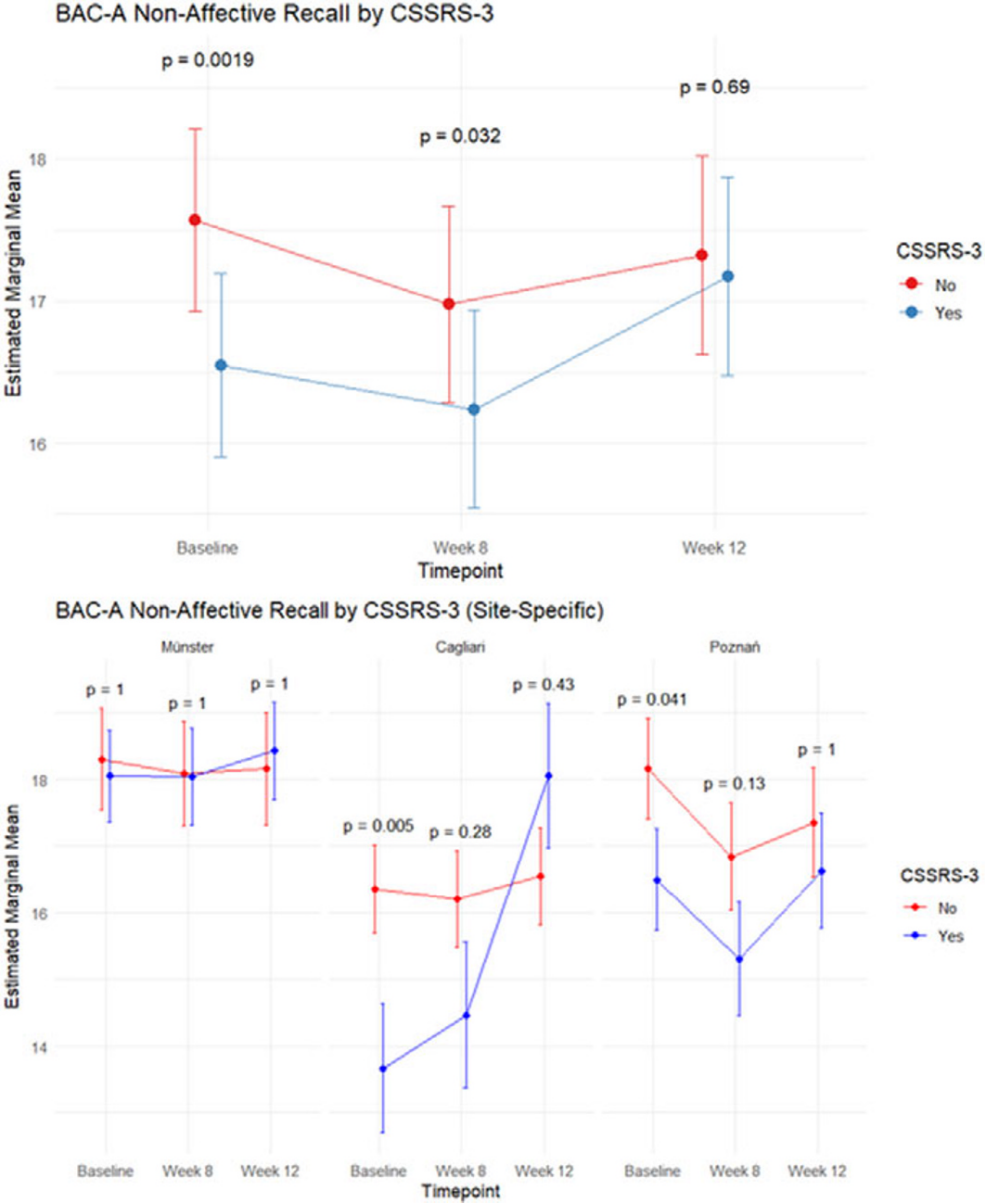
Image 2: long description.

**Image 3:**
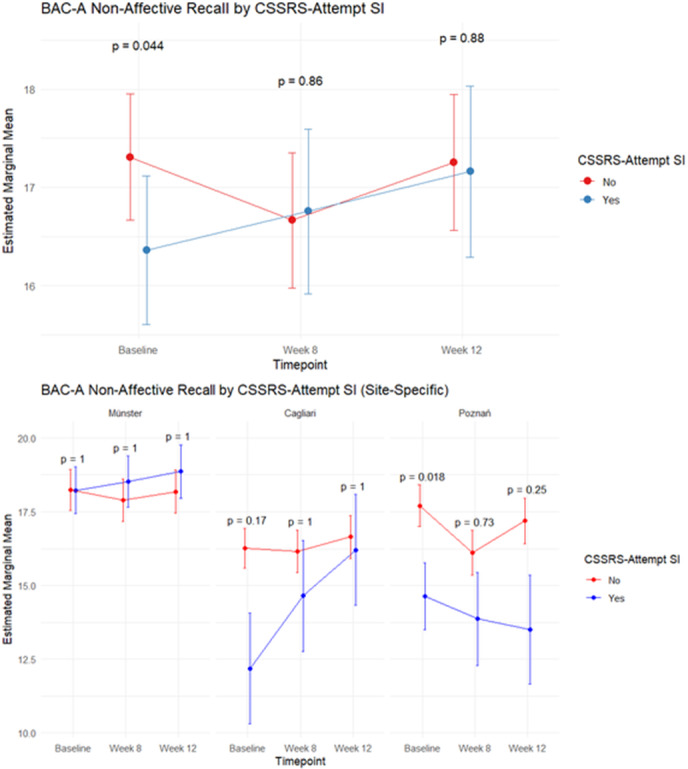
Image 3: long description.

**Conclusions:**

In our sample, impaired non-affective recall appears to represent a stable correlate of lifetime C-SSRS active suicidal ideation in MDD, independent of depression severity and early trauma. Broader cognitive variance was explained by age, education, recruiting site, and treatment factors. These findings support cognitive performance as a potential marker of vulnerability to suicidal behaviour.

**Disclosure of Interest:**

None Declared

## Long descriptions

### Table 1. Long description

The table consists of three columns: Variable, No (n = 174), and Yes (n = 118).

* Age (years), median [I Q R]: No group is 52 [37–62]; Yes group is 41 [29–55].

* Female sex %: No group is 62.6; Yes group is 54.2.

* Education greater than or equal to High School %: No group is 59.0; Yes group is 62.1.

* M A D R S score, median [I Q R]: No group is 22 [19–26]; Yes group is 27 [23–33].

* C T Q total, median [I Q R]: No group is 40 [32–52]; Yes group is 47 [35–59].

* Antipsychotic use %: No group is 26.4; Yes group is 51.7.

* T R D current episode %: No group is 32.2; Yes group is 46.6.

* Recruitment site (Münster/Cagliari/Poznań %): No group is 24.7/52.3/23.0; Yes group is 64.4/9.3/26.3.

Navigate back to Table 1..

### Image 1: Long description

The image consists of two panels.

Top Panel: B A C dash A Non-Affective Recall by C S S R S dash 1.

- Y-axis: Estimated Marginal Mean ranging from 16 to 18.

- X-axis: Timepoint with categories Baseline, Week 8, and Week 12.

- Legend: C S S R S dash 1 No (red) and Yes (blue).

- Data: The red line starts at approximately 17.7 (p = 0.014), dips to 17.1 at Week 8 (p = 0.076), and rises to 17.6 at Week 12 (p = 0.21). The blue line starts at 16.9, dips to 16.5, and rises to 17.1. Error bars indicate confidence intervals.

Bottom Panel: B A C dash A Non-Affective Recall by C S S R S dash 1 (Site-Specific).

- This panel is divided into three sub-plots: Münster, Cagliari, and Poznań.

- Y-axis: Estimated Marginal Mean ranging from 15 to 20.

- Münster: Both red and blue lines hover between 18 and 18.5 across all timepoints with p = 1 at all points.

- Cagliari: The red line stays between 16.5 and 16.8. The blue line starts lower at 15.7 and rises to meet the red line at 16.7 by Week 12. P-values are 0.39, 1, and 1.

- Poznań: The red line starts at 18.2, drops to 16.9, and rises to 17.6. The blue line follows a similar V-shape but lower, starting at 16.9, dropping to 15.4, and rising to 16.8. P-values are 0.32, 0.23, and 1.

Navigate back to Image 1:.

### Image 2: Long description

A multi-panel line graph. The y-axis for all plots is Estimated Marginal Mean and the x-axis is Timepoint with intervals at Baseline, Week 8, and Week 12. A legend indicates red lines for C S S R S dash 3 No and blue lines for C S S R S dash 3 Yes.

Top Panel: B A C dash A Non-Affective Recall by C S S R S dash 3.

- At Baseline, the No group is at approximately 17.6 and the Yes group is at 16.5 (p equals 0.0019).

- At Week 8, the No group drops to 17.0 and the Yes group drops to 16.2 (p equals 0.032).

- At Week 12, the No group rises to 17.3 and the Yes group rises to 17.2 (p equals 0.69).

Bottom Panel: B A C dash A Non-Affective Recall by C S S R S dash 3 (Site-Specific) divided into three sub-plots.

- Münster: Both groups remain stable near 18.0 across all timepoints with p equals 1 at all points.

- Cagliari: The No group stays between 16 and 17. The Yes group starts low at 13.7 (p equals 0.005), rises to 14.5 at Week 8 (p equals 0.28), and peaks at 18.0 at Week 12 (p equals 0.43).

- Poznań: The No group starts at 18.2 and ends at 17.4. The Yes group starts at 16.5 (p equals 0.041), drops to 15.3 at Week 8 (p equals 0.13), and rises to 16.6 at Week 12 (p equals 1).

Navigate back to Image 2:.

### Image 3: Long description

The figure consists of two panels. Both use an X-axis for Timepoint (Baseline, Week 8, Week 12) and a Y-axis for Estimated Marginal Mean. A legend indicates red lines for No and blue lines for Yes regarding C S S R S dash Attempt S I.

Top Panel: B A C dash A Non-Affective Recall by C S S R S dash Attempt S I.

* The No group (red) starts at approximately 17.3 at Baseline, dips to 16.7 at Week 8, and rises to 17.3 at Week 12.

* The Yes group (blue) starts lower at 16.4, rises steadily to 16.8 at Week 8, and reaches 17.2 at Week 12.

* P-values are p equals 0.044 at Baseline, p equals 0.86 at Week 8, and p equals 0.88 at Week 12.

Bottom Panel: Site-Specific data for Münster, Cagliari, and Poznań.

* Münster: Both groups remain stable near 18.0 with p equals 1 at all timepoints.

* Cagliari: The No group (red) is stable around 16.5. The Yes group (blue) starts low at 12.2 and increases sharply to 16.2 by Week 12. P-values range from p equals 0.17 to p equals 1.

* Poznań: The No group (red) fluctuates between 16.0 and 17.5. The Yes group (blue) starts at 14.6 and decreases to 13.5. P-values are p equals 0.018 at Baseline, p equals 0.73 at Week 8, and p equals 0.25 at Week 12.

Navigate back to Image 3:.

